# Efficacy of Short-Term Oral Prednisolone Treatment in the Management of Pericardial Effusion Following Pediatric Cardiac Surgery

**DOI:** 10.1007/s00246-021-02783-y

**Published:** 2021-12-01

**Authors:** Masahiro Mizumoto, Naoki Masaki, Sadahiro Sai

**Affiliations:** Department of Cardiovascular Surgery, Miyagi Children’s Hospital, 4-3-17 Ochiai, Aoba-ku, Sendai, 989-3126 Japan

**Keywords:** Pediatric cardiac surgery, Pericardial effusion, Steroid, Prednisolone, Postpericardiotomy syndrome

## Abstract

A standard treatment for pericardial effusion without cardiac tamponade after pediatric cardiac surgery has not been established. We evaluated the efficacy of short-term oral prednisolone administration, which is the initial treatment for postoperative pericardial effusion without cardiac tamponade at our institution. Between October 2008 and March 2020, 1429 pediatric cardiac surgeries were performed at our institution. 91 patients required postoperative treatment for pericardial effusion. 81 were treated with short-term oral prednisolone. Pericardial effusion was evaluated using serial echocardiography during diastole. Pericardial drainage was performed for patients with circumferential pericardial effusion with a maximum diameter of ≥ 10 mm or signs of cardiac tamponade. Short-term oral prednisolone treatment was administered to patients with circumferential pericardial effusion with a maximum diameter of < 10 mm or localized pericardial effusion with a maximum diameter of ≥ 5 mm. Patients with localized pericardial effusion with a maximum diameter of < 5 mm were observed. Prednisolone (2 mg/kg/day) was administered orally for 3 days, added as needed. Short-term oral prednisolone treatment was effective in 71 cases and 90% of patients were regarded as responders. The remaining patients were deemed non-responders who required pericardial drainage. Overall, 55 responders were deemed early responders whose pericardial effusion disappeared within 3 days. There were no cases of deaths, infections, or recurrence of pericardial effusion. The amount of drainage fluid on the day of surgery was higher in the non-responders. In conclusion, short-term oral prednisolone treatment is effective and safe for treating pericardial effusion without cardiac tamponade after pediatric cardiac surgery.

## Introduction

The incidence of pericardial effusion (PE) after pediatric cardiac surgery has decreased due to advances in perioperative medical care techniques; however, it is still not uncommon. The postoperative PE should be taken care, because it carries the risk of progressing to cardiac tamponade. In the event of accompanying cardiac tamponade, it is generally accepted that pericardial drainage should be indicated [1]. However, in the absence of cardiac tamponade, there is no standard treatment established for postoperative PE, as the preferred treatment differs between various institutions. Generally, several studies have indicated that oral aspirin is often selected as the initial treatment for postpericardiotomy syndrome, whereas additional steroids and colchicine are administered in refractory cases [2–4]. However, there is no consensus regarding the doses to be administered, administration methods, or durations. Furthermore, it takes time for the therapeutic effects to appear. At our institution, short-term oral prednisolone administration is the initial treatment for postoperative PE without cardiac tamponade. In this study, we retrospectively evaluated the effectiveness of this treatment.

## Patients and Methods

**Fig. 1:**
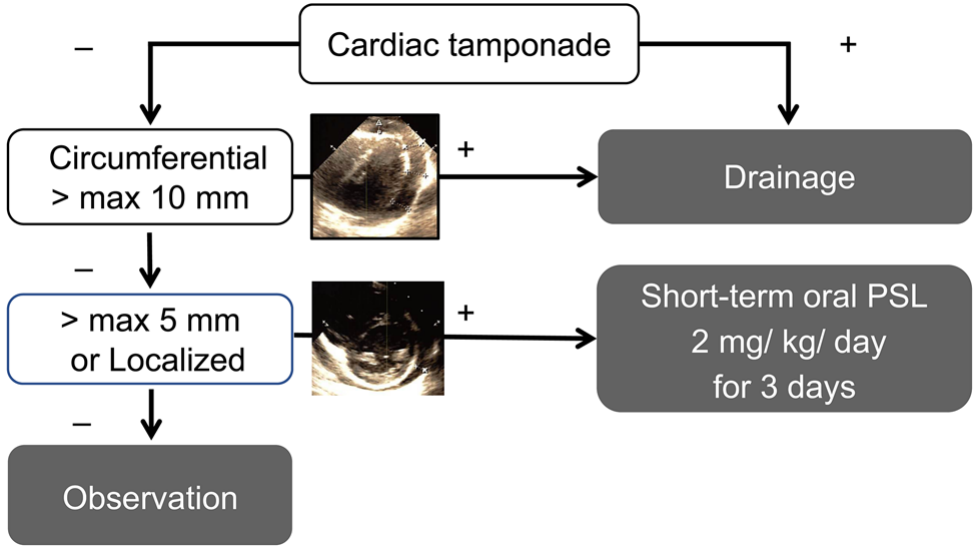
Treatment protocol for PE. *PE* pericardial effusion, *PSL* prednisolone

Between October 2008 and March 2020, 1429 pediatric cardiac surgeries were performed at our institution and postoperative PE requiring therapeutic intervention was observed in 91 patients (6.4%). Among these patients, 81 received short-term oral prednisolone treatment as the first choice. This study was approved by Miyagi children’s hospital ethics committee and all patients provided informed consent. The study subjects, comprising 45 boys and 36 girls, had a median age of 27.0 (11.0–67.5) months and a weight of 10.1 (7.0–16.3) kg. Repeat median sternotomy was performed in 14 of the patients. For all patients, PE was evaluated in the circumferential or localized regions (anterior, lateral, posterior, and inferior) using serial echocardiography, per the short axis, long axis, and four-chamber views in the diastolic phase. The maximum diameter (mm) between the epicardium and pericardium was measured. Figure 1 shows our postoperative PE treatment protocol. Pericardial drainage was indicated in patients with signs of cardiac tamponade or those with circumferential PE with a maximum diameter of ≥ 10 mm. Short-term oral prednisolone treatment was indicated for patients with circumferential PE with a maximum diameter of < 10 mm or localized PE with a maximum diameter of ≥ 5 mm in the absence of signs of cardiac tamponade. In our regimen, prednisolone was administered orally at 2 mg/kg/day, in two divided doses, for 3 days, added as needed. There was no concomitant use with anti-inflammatory drugs (NSAID) such as aspirin or colchicine for postoperative PE. Patients with localized PE with a maximum diameter of < 5 mm were observed. Table 1 shows the main heart diseases and the types of surgery. Ventricular septal defect, tetralogy of Fallot, and atrial septal defect (ASD) accounted for most of the cases. Furthermore, pulmonary artery banding was commonly performed in the palliative surgery. For patients who underwent palliative surgery or for those in whom it was anticipated that subsequent repeat surgery was necessary, a Gore-Tex® sheet (W. L. Gore & Associates, Inc., USA) was placed as a pericardial substitute, while direct closure of the pericardium was performed in the other cases.

**Table 1:** Cardiac diagnosis and type of surgery

Cardiac disease (*n* = 81)		Type of surgery (*n* = 81)	
VSD	24	VSD closure	24
TOF	17	TOF repair	17
ASD	10	ASD closure	10
Valvular disease	8	PAB	8
AVSD	6	Valvular operation	8
PA/VSD	3	Fontan	3
SV	3	mBTS	2
Others	10	Others	9

*ASD* atrial septal defect, *AVSD* atrioventricular septal defect, *mBTS* modified Blalock–Taussig shunt, *PA* pulmonary atresia, *PAB* pulmonary artery banding, *SV* single ventricle, *TOF* tetralogy of Fallot, *VSD* ventricular septal defect

Patients were divided into a responder group, comprising patients whose PE disappeared or significantly decreased with short-term oral prednisolone treatment alone, and a non-responder group, comprising patients who required pericardial drainage after prednisolone treatment. Responders were defined as the disappearance or decrease of localized PE to < 5 mm in all echocardiography, per the short axis, long axis, and four-chamber views. Non-responders were defined as the increase of circumferential PE to > 10 mm in any view of echocardiography or accompanying cardiac tamponade. We analyzed the pre-, intra-, and postoperative risk factors for the non-responders of short-term oral prednisolone treatment. The postoperative blood testing, chest radiography, and echocardiography were performed at 1 week after the surgery.

### Statistical Analyses

JMP 15 (SAS Institute Japan, Tokyo) was used to perform chi-square and Fisher’s exact tests for the statistical analyses. The data are shown as the median ± interquartile range, and a *p* value of < 0.05 was considered significant.

## Results

**Fig. 2:**
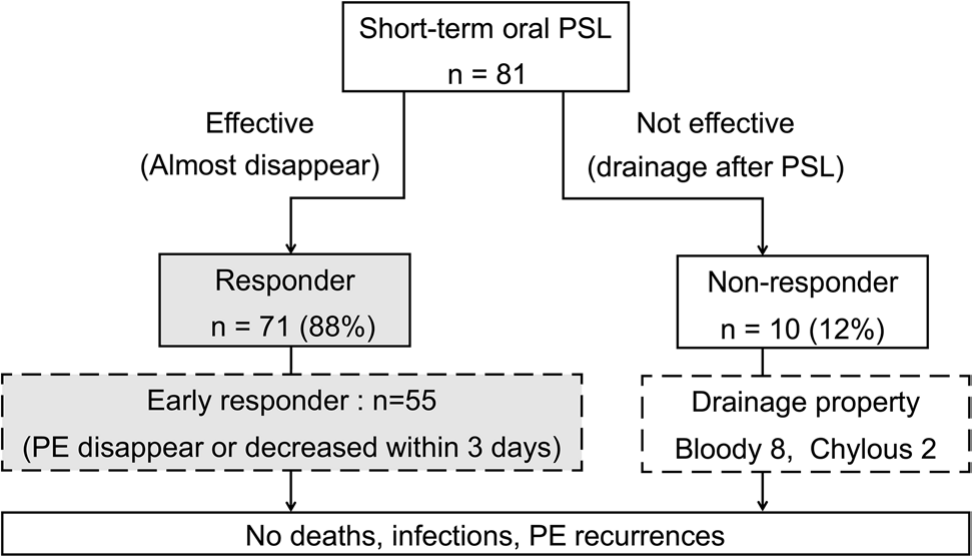
Results of short-term oral PSL treatment. *PE* pericardial effusion, *PSL* prednisolone

**Fig. 3:**
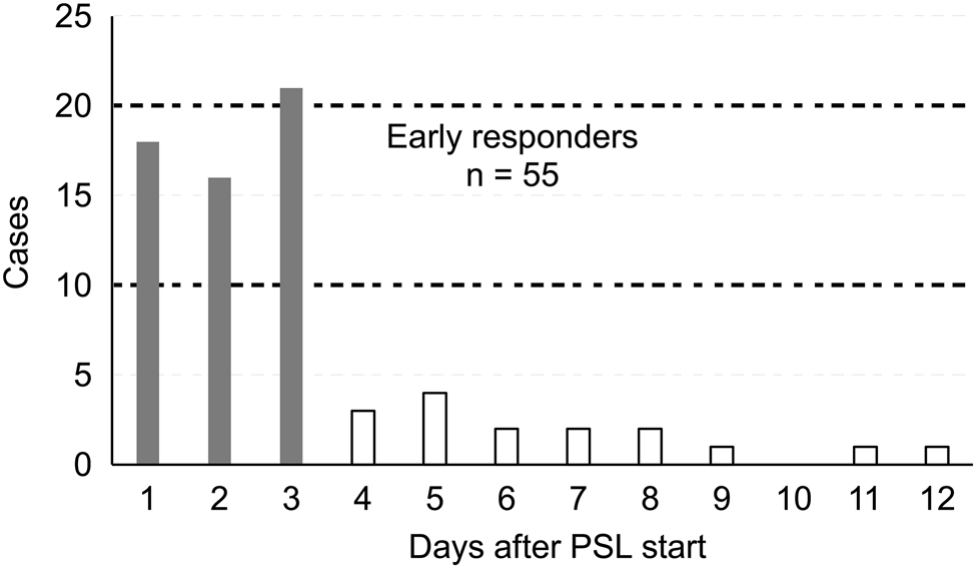
Day of effect on PE decrease in PSL responders. *PE* pericardial effusion, *PSL* prednisolone

There were no deaths, side effects of prednisolone, such as infection, or recurrence of PE. The prednisolone treatment was commenced on postoperative day 8 (8.0–10.0) and the drainage tube after the initial surgery was removed on postoperative day 2.0 (2.0–3.0) in all 81 cases who were administered oral prednisolone. Figure 2 shows the outcomes of the short-term oral prednisolone treatments. In 71 cases, the treatments were effective and approximately 90% of the patients were regarded as responders. Pericardial drainage was required for 10 non-responders. In the non-responder group, the median period of oral prednisolone treatment was 3.0 (1.0–3.0) days, and pericardial drainage was performed immediately after confirmation of increased PE during short-term oral prednisolone treatment. There were no complications from cardiac tamponade and delayed drainage. The property of drained fluid was bloody in eight cases and chylous in two cases. In the responder group, the median period of oral prednisolone treatment and until disappearance of or marked decrease in PE were 3.0 (3.0–3.0) and 3.0 (1.0–3.0) days, respectively. Of these, 55 of the responders were considered as early responders, with a response observed within 3 days from the commencement of the oral prednisolone administration (Fig. 3). Although the remaining 16 responders received additional oral prednisolone, PE was disappeared or significantly decreased without complications or side effects of prednisolone.

Tables 2 and 3 show the analysis of the risk factors of prednisolone non-responders. Among the preoperative factors, the hemoglobin level was higher in non-responders, but there was no significant difference in the frequency of cyanotic heart disease, which can be a cause of polycythemia. Although there were no significant differences between the intra- and postoperative factors, the postoperative cardiothoracic ratio and amount of drainage fluid on the day of surgery tended to be higher in the non-responders than in responders and the inflammatory reaction level tended to be higher in the responders than in non-responders.

**Table 2:** Risk analysis of PSL non-responder characteristics and preoperative factors

Variables	Responder	Non-responder	*p*
	(*n* = 71)	(*n* = 10)	
Age (month)	29.0 (11.0–66.0)	19.5 (9.8–192.8)	0.807
Male/female	37/34	8/2	0.172
Body weight (kg)	10.5 (7.1–16.1)	9.8 (5.4–32.4)	0.858
Previous surgery	12	2	0.681
CTR (%)	54.5 (51.0–59.1)	56.5 (51.0–61.4)	0.486
LVEF (%)	65.4 (56.3–68.4)	69.2 (58.9–73.7)	0.273
Hb (g/dL)	13.5 (12.5–14.3)	15.2 (13.5–16.8)	0.019
BNP (pg/mL)	23.6 (13.2–51.5)	24.3 (19.7–92.9)	0.457
Cardiac disease			0.512
VSD	23	1	
TOF	14	3	
ASD	9	1	
Valvular disease	6	2	
Others	19	3	

*ASD* atrial septal defect, *BNP* brain natriuretic peptide, *CTR* cardiothoracic ratio, *LVEF* left ventricular ejection fraction, *PSL* prednisolone, *TOF* tetralogy of Fallot, *VSD* ventricular septal defect

**Table 3:** Risk analysis of PSL non-responder operative and postoperative factors

Variables	Responder	Non-responder	*p*
	(*n* = 71)	(*n* = 10)	
< Operative factors >			
Emergent or urgent	2	2	0.073
Operation time (min)	275 (182–412)	304 (166–401)	0.88
CPB use	62	8	0.619
CPB time (min)	144 (92–232)	175 (149–233)	0.427
Aorta clamp time (min)	67 (46–125)	108 (88–122)	0.3
Blood transfusion	51	6	0.528
< Postoperative factors >			
CTR (%)	57.0 (53.0–61.0)	61.0 (57.5–66.0)	0.019
Hb (g/dL)	12.9 (11.8–14.4)	13.9 (12.9–15.1)	0.151
CRP (mg/dL)	2.56 (0.77–3.10)	1.87 (0.20–3.23)	0.113
BNP (pg/mL)	117 (85–224)	192 (102–372)	0.35
Drainage output (mL/kg/h)			
(First 4 h)	1.20 (0.61–2.15)	1.80 (0.98–3.89)	0.087

*BNP* brain natriuretic peptide, *CPB* cardiopulmonary bypass, *CTR* cardiothoracic ratio, *PSL* prednisolone

## Discussion

In this study, we followed our institution’s treatment protocol for PE after cardiac surgery and demonstrated that the administration of oral prednisolone at a dose of 2 mg/kg/day for 3 days results in good outcomes, as indicated by the 90% response rate with no deaths or complications. In addition, its effect was observed in the short term, as the median period until the disappearance of or marked decrease in PE after the prednisolone treatments began was 3.0 days.

The incidence of PE after congenital heart disease surgery has declined due to advances in perioperative medical care. In the 1980s, the PE rate was 53–65% [5–7], but it declined to 13.6–23.0% in the 1990s [8–10] and to 10% in recent years [11, 12]. Despite the consistent decline of the PE rate, postoperative PE remains a serious complication that can cause cardiac tamponade. In this study, the incidence of PE requiring treatment after pediatric cardiac surgery was 6.4%. Pericardial drainage is indicated for patients with signs of cardiac tamponade. However, the standard treatment for PE in patients who have no symptoms of cardiac tamponade after cardiac surgery differs between institutions. In general, postpericardiotomy syndrome is considered a potential cause of PE; therefore, oral aspirin or NSAID are often selected as the initial treatment. However, the use of aspirin has some shortcomings as its therapeutic effect is variable and it takes time for PE to decrease [10, 11]. Additionally, oral aspirin treatment has been reported to have no prophylactic effect on postoperative PE [7]. In refractory cases, additional administration of steroids or colchicine can be considered [2–4]. Only a few reports have examined any details of steroid treatment for PE after pediatric cardiac surgery, such as the type of drug to use or the method and duration of administration [11, 12]. Reports on the use of colchicine for postoperative PE often describe its prophylactic use and treatment of recurrence in adults [3, 13–18]. However, its safety and efficacy in children are unclear [12]. Furthermore, colchicine therapy is often administered over several months and in conjunction with oral NSAID (aspirin and ibuprofen) and prednisolone. Therefore, its clinical use in children is considered arduous.

Dalili et al. [11] reported that oral aspirin treatments of 50–70 mg/kg/day (divided into four doses) resulted in a response rate of 77%, with a mean time to PE disappearance of 13 ± 8 days, whereas oral prednisolone treatments of 1 mg/kg/day (divided into four doses) resulted in a response rate of 90%, with a mean time to PE disappearance of 7 ± 3 days. The response rate in the current study was equivalent to these results; however, it is likely that our treatment protocol was more useful because patients in the current study only received two doses of oral prednisolone per day and its therapeutic effects were observed in the shorter term. For proper drug compliance in children, it is important for clinicians to consider a shorter term of treatment and smaller number of doses per day. Shorter-term treatments may also lead to reduced hospital stays.

Alternatively, it should be noted that there were 10 non-responders who required pericardial drainage. Various risk factors for PE after pediatric cardiac surgery have been reported, such as advanced age, large physique, female sex, trisomy 21, ASD surgery, the Fontan procedure, cardiopulmonary bypass, postoperative warfarin administration, large amounts of postoperative fluid drainage, and previous cardiac surgery [10–12, 19]. Considering these variables, we conducted a prednisolone unresponsiveness risk factor analysis using patient background and pre-, intra-, and postoperative factors. Although the patient background and pre- and intraoperative factors were not significant indicators of the ineffectiveness of prednisolone, among the postoperative factors, the cardiothoracic ratio was significantly higher and the amount of drainage fluid on the day of surgery tended to be higher in non-responders. The fluid drained was bloody in eight of the 10 non-responders; therefore, a large amount of bloody drainage fluid on the day of surgery might be a predictor of postoperative PE and non-responsiveness to short-term prednisolone treatment.

This study has some limitations. First, the sample size was small. Based on our encouraging results, further studies with a larger number of subjects are needed. Second, our protocol did not specify in detail when to commence short-term oral prednisolone treatments. Furthermore, routine postoperative echocardiography was performed 1 week after surgery; therefore, short-term oral prednisolone treatment was started on postoperative day 8 in most cases. In previous studies [10, 11], the mean timing of the PE diagnosis was reported to be on postoperative day 11 and most cases were diagnosed within 2 weeks after cardiac surgery. Thus, the commencement of short-term oral prednisolone treatment on postoperative day 8 in this study might have contributed to the high-treatment response rate. Although earlier postoperative treatments could have been more effective, the appropriate timing of the treatments should be carefully examined. Prophylactic steroid use for postpericardiotomy syndrome was ineffective in a previous study [20]. In addition, attention should be paid to adverse reactions to steroid use in the early postoperative phase.

## Conclusion

In this study, short-term oral prednisolone administration for the treatment of PE with no signs of cardiac tamponade after pediatric cardiac surgery showed a notable response rate of 90% with an earlier therapeutic effect, indicating that it is a safe and effective novel treatment.

## Data Availability

All data and materials, as well as software application, support our published claims and comply with field standards.
